# A Structure of a Collagen VI VWA Domain Displays N and C Termini at Opposite Sides of the Protein

**DOI:** 10.1016/j.str.2013.06.028

**Published:** 2014-02-04

**Authors:** Ann-Kathrin A. Becker, Halina Mikolajek, Mats Paulsson, Raimund Wagener, Jörn M. Werner

**Affiliations:** 1 Center for Biochemistry, Medical Faculty, Center for Molecular Medicine Cologne (CMMC), Cologne Excellence Cluster on Cellular Stress Responses in Aging-Associated Diseases (CECAD), University of Cologne, Cologne 50931, Germany; 2 Centre for Biological Sciences, B85 Life Sciences Building M55, University of Southampton, Southampton SO17 1BJ, UK

## Abstract

Von Willebrand factor A (VWA) domains are versatile protein interaction domains with N and C termini in close proximity placing spatial constraints on overall protein structure. The 1.2 Å crystal structures of a collagen VI VWA domain and a disease-causing point mutant show C-terminal extensions that place the N and C termini at opposite ends. This allows a “beads-on-a-string” arrangement of multiple VWA domains as observed for ten N-terminal domains of the collagen VI α3 chain. The extension is linked to the core domain by a salt bridge and two hydrophobic patches. Comparison of the wild-type and a muscular dystrophy-associated mutant structure identifies a potential perturbation of a protein interaction interface and indeed, the secretion of mutant collagen VI tetramers is affected. Homology modeling is used to locate a number of disease-associated mutations and analyze their structural impact, which will allow mechanistic analysis of collagen-VI-associated muscular dystrophy phenotypes.

## Highlights

• The structure of a VWA domain (N5) of collagen VI at 1.2 Å is presented
• N and C termini of the domain are at opposite ends
• The structure with a myopathy-causing mutation shows altered interaction interface
• The impact of mutations in collagen VI VWA domains was analyzed

## Teaser

Becker et al. describe a crystal structure of a von Willebrand factor A (VWA) domain, which has a C-terminal extension that places the N- and C-termini at opposite ends not seen before for A domains. This allows a “beads-on-a-string” arrangement of multiple VWA domains as observed for the collagen VI α3 chain.

## Introduction

Von Willebrand factor A (VWA) domains consist of about 200 amino acid residues and act as interaction modules in many intra- and extracellular proteins, e.g., in copines, integrins, von Willebrand factor, complement factors B and C2, matrilins, and collagens (for review, see Whittaker and Hynes, 2002). They adopt a Rossmann fold with a central β sheet surrounded by amphipathic α helices (Rossmann et al., 1974). The N- and C-termini are close to another and the structure is often stabilized by two terminal cysteine residues that form a disulfide bridge. VWA domains often contain a highly conserved metal-ion-dependent adhesion site (MIDAS) motif that is involved in ligand binding (Lee et al., 1995).

The structures of VWA domains in several proteins have been determined in the past. Among these, the integrin I domains, the VWA domains of the von Willebrand factor, and the complement factor VWA domains are best characterized (for review, see Springer, 2006). On the basis of such structures, the role of the MIDAS motif in ligand binding has been studied in detail. In integrin I domains, the MIDAS motif can exist in different activation states with varying affinities to the ligands. In addition, a conformational change of the I-domain occurs upon ligand binding in which the C-terminal α7 helix moves axially toward the C terminus. Such structural changes have so far not been described for any other VWA domain. Some VWA domains, e.g., the von Willebrand factor A3 domain, bind their ligands via binding sites other than the MIDAS motif (Bienkowska et al., 1997, Romijn et al., 2001). No structure of a VWA domain from the collagen phylogenetic branch has yet been solved. Eight of the 28 known collagens carry VWA domains (collagen VI, VII, XII, XIV, XX, XXI, XXII, and XXVIII; for review, see Gordon and Hahn, 2010). Indeed, a large proportion of the VWA domains present in mouse proteins occur in collagens. The six collagen VI α chains contain 46 VWA domains, and these are often involved in ligand binding (Bonaldo et al., 1990, Specks et al., 1992, Wiberg et al., 2003).

**Figure 1:**
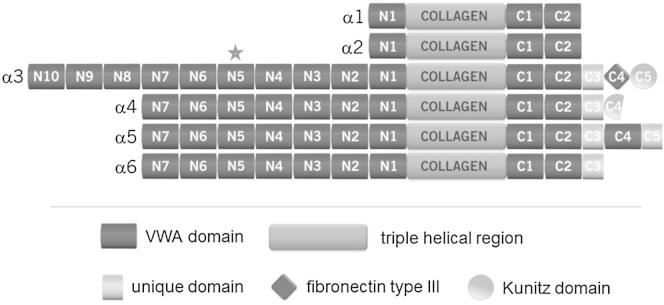
Domain Structure of the Collagen VI Chains The numbering of the domains represents the order of the N- and C-terminal domains in each chain. The asterisk indicates the N5 domain of the α3 chain.

Collagen VI is a ubiquitously expressed extracellular matrix protein that forms a structurally unique network of beaded microfilaments. It is expressed in almost all connective tissues, often in association with basement membranes. For a long time, collagen VI was thought to consist of only three chains: α1, α2, and α3. Recently, three novel chains (α4, α5, and α6) were identified that are highly homologous to the α3 chain (Fitzgerald et al., 2008, Gara et al., 2008). All collagen VI chains consist of a relatively short triple-helical region as well as of N- and C-terminal globular structures that are largely made up of VWA domains (Figure 1). The N termini of the shorter α1 and α2 chains contain one VWA domain each, whereas the longer α3, α4, α5, and α6 chains are composed of ten or seven N-terminal VWA domains. At the C terminus, all chains contain two or, in case of the α5 chain, three VWA domains and additional domains.

Collagen VI microfibril formation has been studied in molecules made up by the classical chains (Baldock et al., 2003, Beecher et al., 2011, Engel et al., 1985, Furthmayr et al., 1983). In a stepwise assembly, the α1, α2, and α3 chains form triple-helical monomers that then assemble into disulfide-bonded antiparallel dimers, followed by the alignment of two dimers to antiparallel tetramers that are also stabilized by disulfide bonds. After secretion, the tetramers form microfibrils by connecting in a head-to-head-fashion, resulting in a “beads-on-a-string” appearance. The novel chains are believed to substitute for the α3 chain (Gara et al., 2008). The C-terminal collagen VI domains are important for assembly and microfibril formation (Ball et al., 2001, Lamandé et al., 2006, Tooley et al., 2010), but the N-terminal N1–N5 domains have also been shown to be critical for collagen VI suprastructure (Fitzgerald et al., 2001).

Mutations in the *COL6A1*, *COL6A2*, and *COL6A3* genes lead to the musculoskeletal diseases Bethlem myopathy (BM) and Ullrich congenital muscular dystrophy (UCMD); (for review, see Allamand et al., 2011, Lampe and Bushby, 2005). BM and UCMD represent the mild and the severe end, respectively, of one clinical spectrum whose hallmarks include proximal muscle weakness; variable contractures mostly affecting the long finger flexors, elbows, and ankles; and unusual skin features such as hypertrophic scars or keloid formation. In addition, UCMD patients show early-onset muscle weakness, hyperelasticity of distal joints, congenital hip dislocations, and, with progression of the disease, spinal rigidity, scoliosis, and respiratory failure. Mostly, BM is caused by recessive and UCMD by dominant mutations. However, putative recessive mutations have also been described for UCMD patients (Lampe et al., 2005). Mutations causing BM are associated with reduced collagen VI levels, and in UCMD a complete loss of collagen VI is often caused by introduction of premature stop codons (Baker et al., 2005). Missense mutations causing UCMD have also been described, although the disease mechanism is unclear (Lampe et al., 2005).

To date, the Leiden open variation database (LOVD; http://www.dmd.nl) reports 35 missense mutations in the exons encoding the N-and C-terminal VWA domains of the collagen α3 chain. One of these missense mutations, R1064Q in human (R1064Q^h^) that is located in the N5 domain, was found in an UCMD patient (Lampe et al., 2005). This domain has also been shown to be essential for collagen VI microfibril assembly (Fitzgerald et al., 2001). To gain insight into the disease-causing mechanism, we crystallized both the wild-type murine collagen VI α3N5 domain and the N5 domain carrying the R1061Q mutation (R1061Q^m^), which corresponds to human R1064Q^h^. We studied the impact of the mutation on the secretion of the single VWA domain and the secretion and assembly of collagen VI tetramers. The structural information was used to model other collagen VI VWA domains and to evaluate the impact of known mutations on the domain structures, thereby providing a basis for genotype-phenotype analysis.

## Results and Discussion

### Structure Determination of the Collagen VI α3N5 Domain

**Figure 2:**
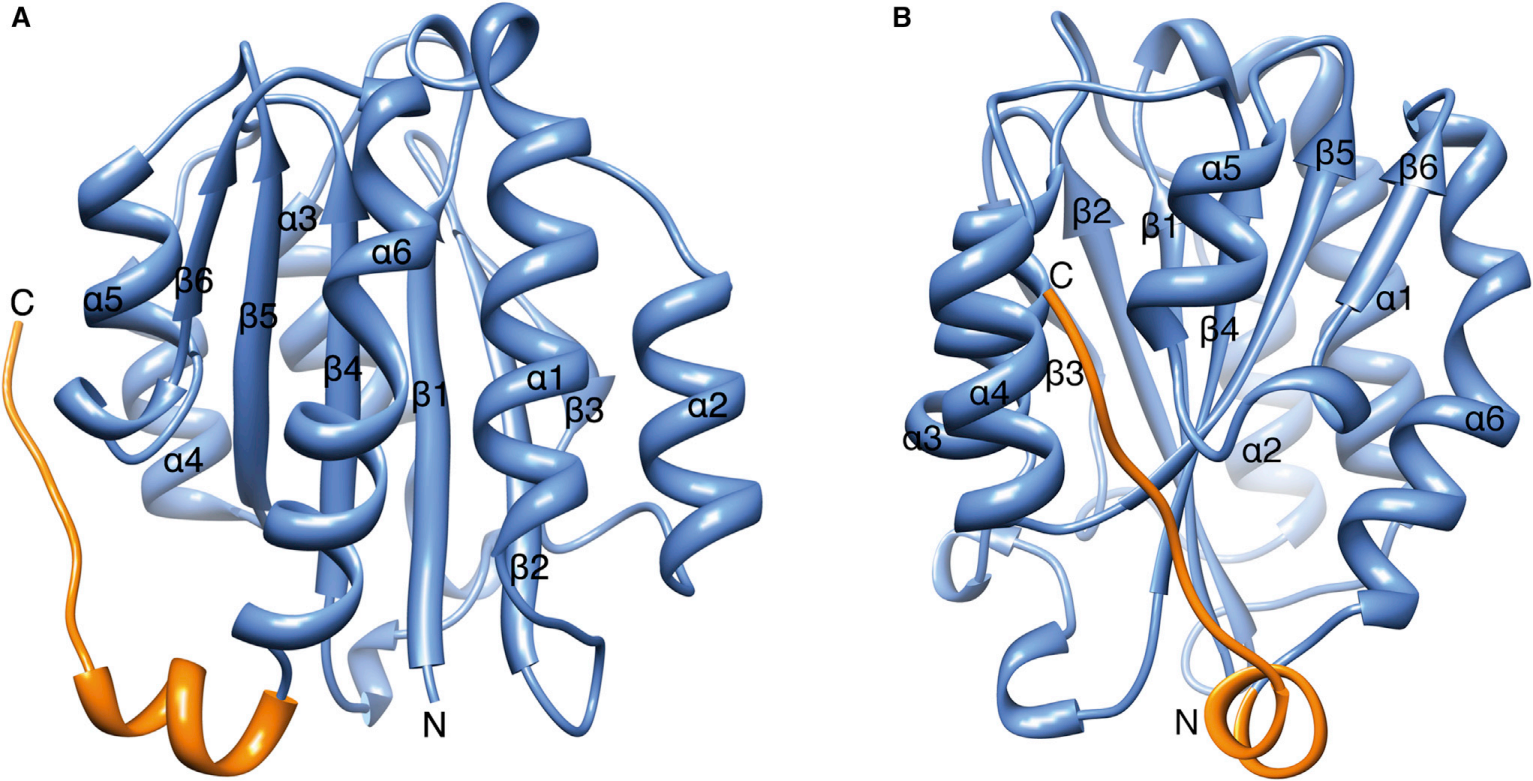
Structure of Collagen VI α3N5 (A) Ribbon diagram of the murine collagen VI α3N5 domain. β strands and α helices of the VWA fold are labeled β1 to β6 and α1 to α6, respectively, and are colored in blue. The C-terminal extension (residues N1206-G1224) is shown in orange. (B) Same as (A) but rotated by 90°.

**Figure 3:**
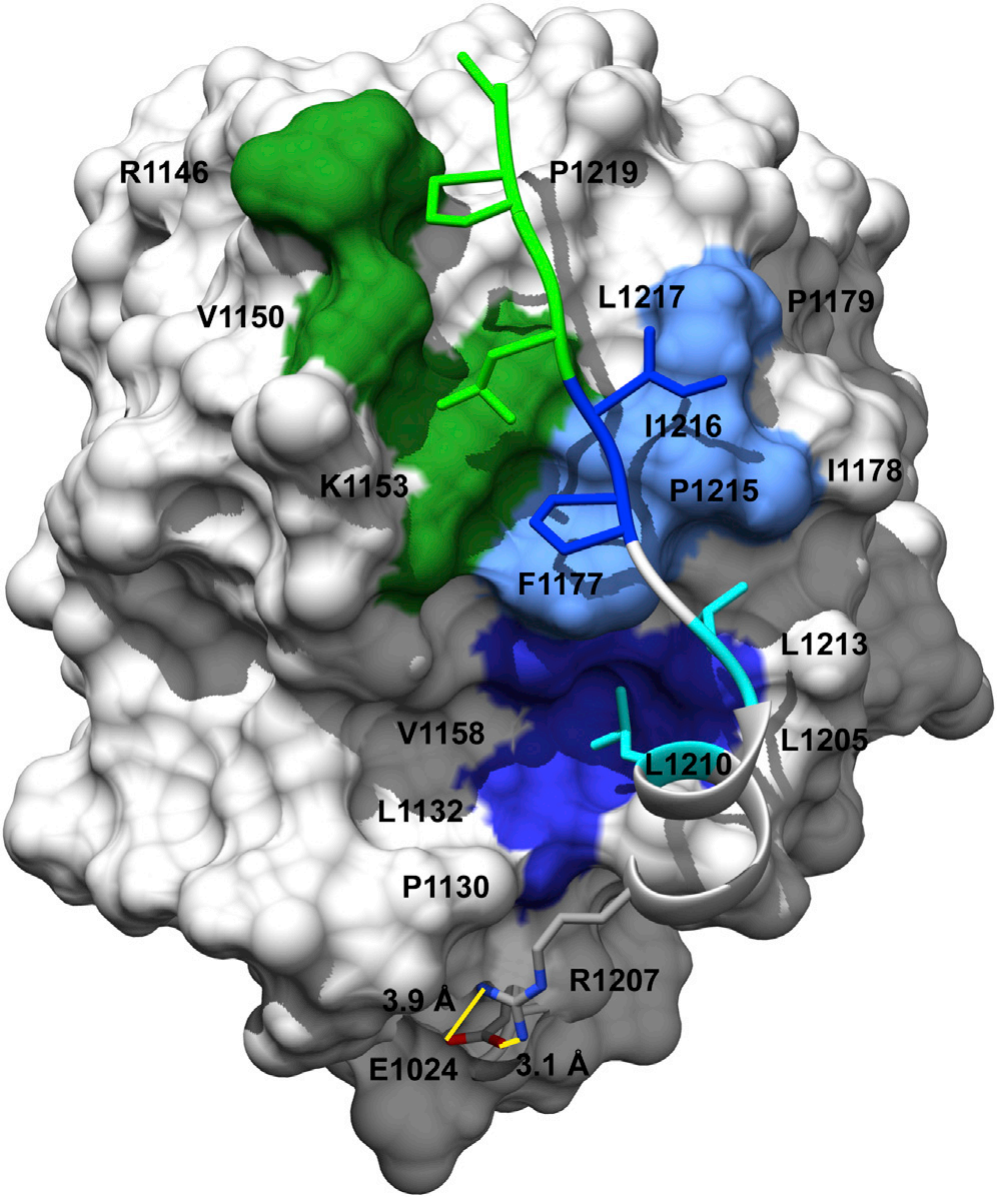
Analysis of the C-Terminal Extension Surface representation of the murine collagen VI α3N5 domain combined with a ribbon representation of the C-terminal extension showing the residues at the interface with the core domain. The two regions on the core domain that form hydrophobic contacts with the C-terminal extension are shown in dark blue and light blue, respectively. Residues in the extension are shown as ball and sticks. The region that forms contacts with the C-terminal extension via a ladder is shown in green. The residues forming the salt bridge are presented as ball and sticks (gray). The lines (yellow) represent the distances in Angstrom.

The murine collagen VI α3N5 domain was crystallized and its structure solved by molecular replacement at 1.2 Å resolution (Table 1). The crystal asymmetric unit contains one molecule of collagen VI α3N5 comprising residues E1024–P1219. For the remaining five residues, S1220–G1224, poorer electron density precluded unambiguous model building. The structure has a central six-stranded hydrophobic β sheet flanked on either side by three amphipathic α helices (Figure 2). Based on the nomenclature of the von Willebrand factor A2 (vWFA2) domain (Zhang et al., 2009), β strands and α helices are labeled β1 to β6 and α1 to α6, respectively. Similarly to other members of the VWA fold, the α4 helix is well formed, while in the vWFA2 domain the α4 helix is absent (Jakobi et al., 2011, Zhang et al., 2009). Like most of the collagen VI VWA domains, the α3N5 domain does not contain a conserved MIDAS motif. The collagen VI α3N5 domain structure is the first solved structure of a collagen VWA domain and differs from other VWA domains in having an extended C-terminal region comprising residues N1206–G1224 (Figure 2). The C-terminal extension comprises a short α helix approximately at right angles with helix α6 followed by an extended region that cradles between the α4 and α5 helices of the N5 domain (Figure 2B). Closer examination of the structure in this region shows that the extension is linked to the core domain by a salt bridge formed by residue R1207 from the C-terminal extension and E1024 from the core domain (Figure 3). Furthermore, residues L1210 and L1213 pack into a hydrophobic patch (patch 1; Figure 3, dark blue) formed by the residues P1130, L1132, V1158, and L1205 from the core domain, and a second hydrophobic patch (patch 2; Figure 3, light blue) is created by the side chains F1177, I1178, and P1179 from the core domain, which is buried by P1215 and I1216 from the C-terminal extension (Figure 3). In addition, residue I1216 also forms a hydrogen bond to the backbone carbonyl oxygen of residues I1178. The last two residues of the extension showing unique electron density, residues L1217 and P1219, interact with a hydrophobic ladder created by the long hydrophobic portions of the side chains of residues R1146, V1150, and K1153 of the α4 helix (Figure 3, green). As a result, the N and C termini are at opposite sides of the domain, which allows the formation of beads-on-a-string assemblies of multiple domains. All previously reported VWA domains have their N and C termini in close proximity at the same side of the protein. For a number of VWA domains, N- and C-terminal cysteine residues form disulfide bridges that close and stabilize the structure.

**Table 1:** Data Collection and Refinement Statistics for Collagen VI α3N5 and R1061Q Mutant

	Collagen VI α3N5	Collagen VI α3N5 R1061Q
**Data Collection**		

Space group	P2_1_	P4_3_2_1_2
Unit cell dimensions (Å)	a = 37.7	a and b = 55.1
	b = 58.6	
	c = 39.3	c = 106.9
Resolution (Å)	36 - 1.2 (1.26-1.2)	38 - 1.2 (1.22-1.2)
No of molecules in asymmetric unit	1	1
No. of reflections	114,428 (14214)^a^	353,145 (12,132)
No. of unique reflections	47,672 (6,693)	53,401 (3,739)
Multiplicity	2.4 (2.1)	6.6 (3.2)
Completeness (%)	97 (93.8)	99.7 (96.6)
R_merge_ (%)^b^	4.5 (17.4)	4 (49)
<I/σ(I) >	10.5 (4.5)	19.3 (2.2)

**Refinement**		

No. of reflections for the work-data set^c^	45,221	50,607
No. of reflections for the test-data set^b^	2,414	2,707
R/R_free_ (%)	13.57/16.19	13.88/16.61
No. of atoms protein/water	3,258/236	3,214/215
Rmsd bond length (Å)	0.009	0.009
Rmsd bond angles (°)	1.34	1.32
B factors (Å^2^)		
Protein	14.6	17.7
Waters	29	31.5
For all atoms	15.6	18.6

a Values in parenthesis are for the highest-resolution shell.

b R_merge_ = ∑_hkl_ ∑_i_|I_i_-I|/∑_hkl_∑_i_|I_i_|, where I_i_ and I are the i^th^ and mean measure of the intensity of reflection hkl.

c R_work_ = ∑_hkl_ ||F_obs_| -|F_calc_||/∑_hkl_|F_obs_|, where F_obs_ and F_calc_ are the observed and calculated structure factors, respectively. R_free_ is calculated as for R_work_ but from a subset of the data (5%) that were withheld.

The C-terminal extension observed here allows a near-linear arrangement of the adjacent VWA domain in the collagen VI α3 chain. This is in good agreement with the domain arrangement in collagen VI (Figure 1) and the observed extended structure of the N9-N1 fragment of collagen VI α3 (Beecher et al., 2011). Analysis of the Cα atom B factors shows that the C-terminal extension is less ordered than the core of VWA domain (data not shown), suggesting a degree of plasticity in the arrangement of the N5-N4 domain pair.

### Analysis of the C-Terminal Extension in Tandem VWA Domains

Regions flanking folded domains are well placed to regulate domain function by interacting with the adjacent core domain and/or by constraining the overall architecture of the intact protein and its complexes. We created homology models of the α3N2-N10 domains to identify common features of the extended C-terminal region. The modeled structures are very similar leading to pairwise root-mean-square deviations (rmsds) of less than 0.6 Å with the α3N5 domain. The largest deviations result from three- or four-amino acid insertions in the α3N7 and α3N10 domains located in the loop prior to the β6 strand and the α6 helix, respectively. The resulting structures were analyzed for conserved features of the interactions between the core domains and the C-terminal extensions that were seen in the α3N5 domain (Figure 3). The interactions between hydrophobic patch 1 and the C-terminal extension are well conserved for the α3N2-N9 domains and in α3N10 hydrophobic interactions are replaced by charge interactions due to concomitant residue changes in the core domain and the extension. In contrast, the residues creating patch 2 are not well conserved. Together with the sequence variations in the C-terminal extension facing patch 2, this suggests that the observed interactions in α3N5 are not maintained among the α3N2–N10 domains beyond the region defined by patch 1.

**Figure 4:**
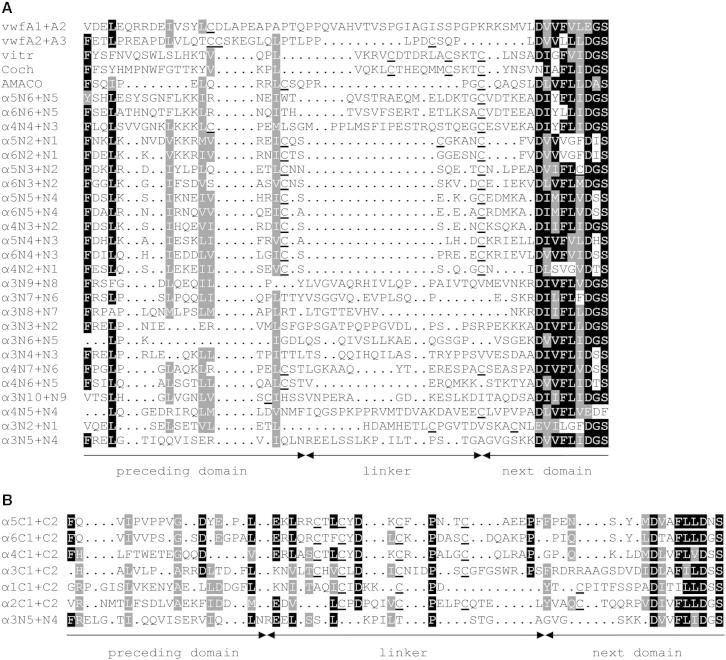
Amino Acid Sequence Alignment of the Linker Regions of Tandem VWA Domains in Mouse The sequences were from the ENSEMBL database and aligned by the Pileup program. (A) Alignment of the linkers of von Willebrand factor (vwf), vitrin (vitr), cochlin (coch), and the N-terminal domains of collagen VI α3, α4, α5, and α6. The domains are indicated by N10-N1. (B) Alignment of the collagen VI α3N5-N4 linker and the linkers of the C1 and the C2 domains of all six collagen VI chains. Cysteine residues are underlined. Below, the borders between the linker and the adjacent domains are indicated. Note that the α5N7-N6 and α6N7-N6 could not be aligned to any other linker.

To further analyze the C-terminal extension, sequence alignments of tandem VWA domains were performed (Figure 4). In murine proteins, there are 38 tandem VWA domains that are predominantly found in collagen VI. In addition, von Willebrand factor has two and vitrin, cochlin, and AMACO have one tandem VWA domain each. The alignments show that the linkers are highly diverse, probably reflecting the specific structural requirements of each connection. A good example of this diversity is the lack of similarity between the equivalent collagen VI α5N7-N6 and the collagen VI α6N7-N6 linker and other linkers. Thus, a comprehensive alignment was not possible, in particular because of the differing sequences of the C-terminal VWA domains of the collagen VI chains. Previous phylogenetic analysis has shown that those VWA domains group to a distinct branch (Gara et al., 2008). Hence, they were aligned separately with the collagen VI α3N5-N4 linker region (Figure 4B). A unique feature of the C-terminal domains is the presence of cysteine residues, which may be involved in interchain crosslinking of the collagen VI chains. Interestingly, a single cysteine residue is also present in the linkers of vitrin and cochlin and in the linker between the N10 and the N9 domains and between the N2 and the N1 domains of the collagen VI α3 chain and between the N2 and the N1 domains of the collagen VI α5 chain. The length of the linkers varies greatly, with the longest linker occurring between the A1 and the A2 domain of the von Willebrand factor and the shortest in AMACO. It is therefore not surprising that direct interactions between two tandem VWA domains have been described only for the A1 and the A2 domain of the von Willebrand factor (Martin et al., 2007). It is also evident that the linkers between the N-terminal domains of the classic α3 chain have similar lengths, while those in the collagen VI α4, α5, and α6 chains are mostly shorter. This may suggest that their N-terminal string of VWA domains is more compact. Interestingly, in contrast to the α3 chain, the N-terminal VWA domains of the α4, α5, and α6 chains mostly possess cysteine residues at the N- and the C terminus which are known to form long range disulfide bonds in other VWA domains that may restrain their structure.

### Crystal Structure of the Mutated Collagen VI α3N5 Domain R1061Q^m^

**Figure 5:**
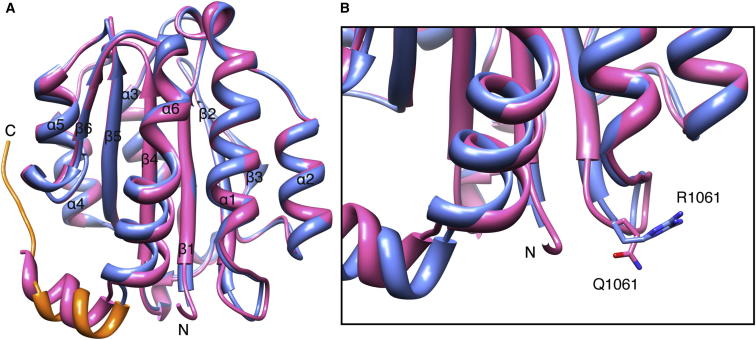
Comparison of Wild-Type and R1061Q^m^ Mutant Collagen VI α3N5 Domains (A) Ribbon diagram of wild-type (blue, C-terminal extension in orange) and R1061Q^m^ mutant structure (pink). (B) Expansion of (A) around the location of the mutation. The side-chain conformations of arginine and glutamine in position 1061 are shown in blue and pink stick, respectively.

To gain insight into the UCMD causing mechanism of the R1064Q mutant in human, the equivalent mutation R1061Q^m^ in mouse was inserted into the murine collagen VI α3N5 domain by mutagenesis PCR and the crystal structure of the corresponding recombinant protein was determined at 1.2 Å resolution (Table 1). The overall structure of the mutant VWA domain is very similar to the wild-type collagen VI α3N5 domain (Figure 5A) with an rmsd of 0.54 Å. The most significant structural difference is around the C terminus where the α6 helix of the mutant has a small angle with respect to the wild-type leading to a 1.5 Å displacement of the helix in the C-terminal extension. Poor electron density after residue L1213 in the mutant precluded the unambiguous assignment of the remainder of the C-terminal extension residues K2014–G2024.

The residue of the R1061Q^m^ mutation is preceding the beginning of the β2 strand and is located near the N terminus. Comparison of the wild-type and mutant structures shows that the side chains of the wild-type arginine and mutant glutamine are surface exposed and are pointing in approximately opposite directions while the remainder of the local conformation is the same (Figure 5B). The crystal packing of the R1061Q^m^ collagen VI α3N5 domain is different from the wild-type. While the arginine residue in the wild-type is in close proximity to the α2α3 loop of the neighboring molecule, the glutamine residue of the R1061Q^m^ mutant interacts with the C-terminal part of the adjacent molecule (data not shown). The difference in crystal packing is likely to contribute to the differences between wild-type and mutant structures in the C-terminal extension. The charge difference in the mutant and the difference in crystal packing also indicate the possibility that this mutation may alter intra- or intermolecular interactions and therefore disturb the assembly of collagen VI. Such surface exposed mutations differ from mutations in the core of VWA domain structures, e.g., the many mutations in the matrilin-3 VWA domain that cause multiple epiphyseal dysplasia (MED) due to endoplasmic reticulum (ER) stress and the unfolded protein response (Cotterill et al., 2005, Leighton et al., 2007, Nundlall et al., 2010, Otten et al., 2005).

### Impact of the R1061Q^m^ Mutation on Secretion and Assembly of Collagen VI

**Figure 6:**
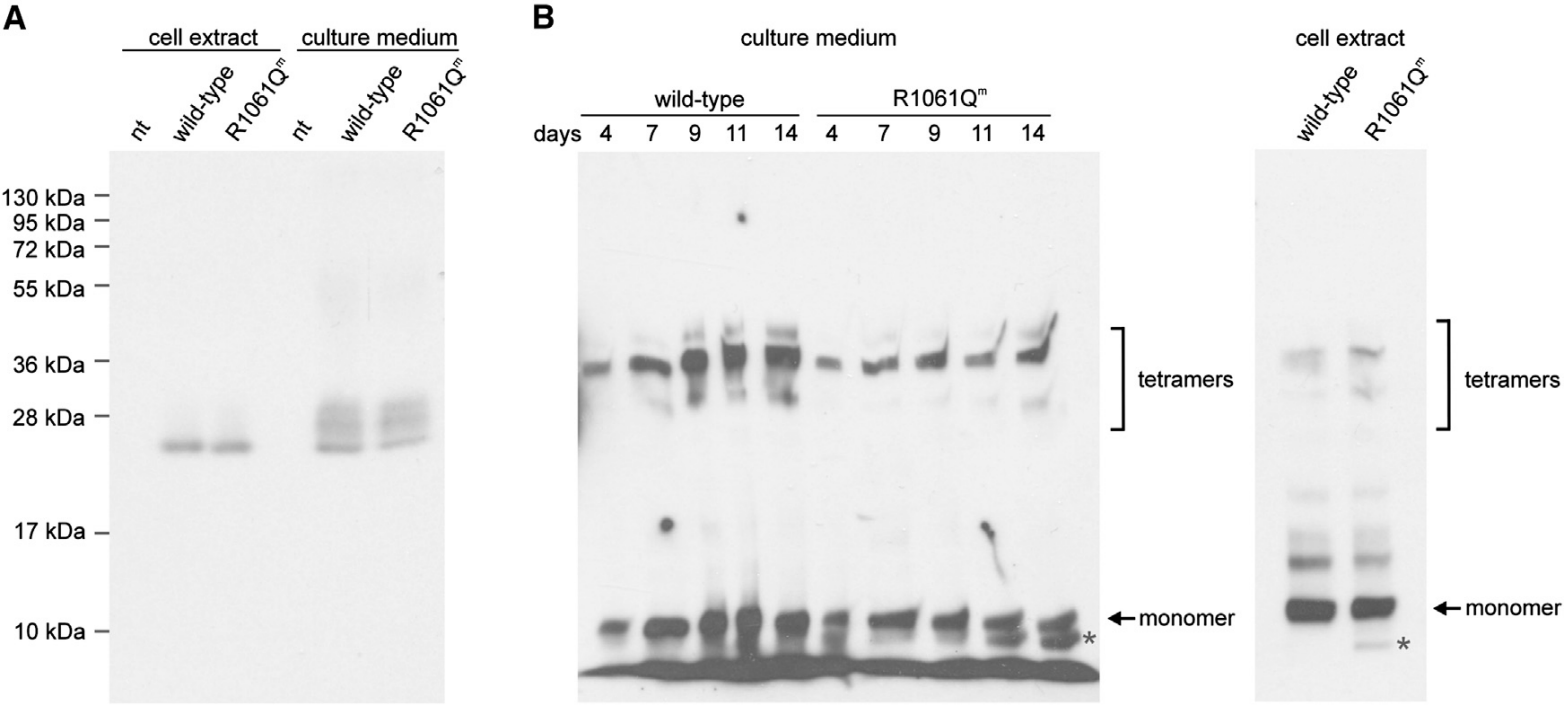
Immunoblot Analysis of Effects of the R1061Q^m^ Mutation on Secretion and Collagen VI Assembly (A) Untransfected 293EBNA cells (nt) and cells transfected with the wild-type collagen VI α3N5 domain or the N5 domain carrying the R1061Q^m^ mutation were cultured for 2 days after transfection. Culture medium and cell extracts were harvested and subjected to electrophoresis on a 12% SDS-polyacrylamide gel under reducing conditions. The α3N5 domain was detected with a polyclonal antibody against the strep tag. The molecular masses of marker proteins are indicated on the left. (B) SaOS-2 cells stably transfected with the wild-type collagen VI α3 chain (N9-C5) or the α3 chain carrying the R1061Q^m^ mutation were cultured for up to 14 days. Culture medium was harvested after 4, 7, 9, 11, or 14 days. After 14 days, the cell layer was extracted. Culture medium and cell extracts were analyzed by electrophoresis on 0.5% agarose/2.4% polyacrylamide composite gels after treatment with 2M urea under nonreducing conditions. α3-chain-containing proteins were detected with a polyclonal antibody against the α3 C terminus. Collagen VI monomers and tetramers are indicated on the right. The sizes were estimated from purified collagen VI protein (Gara et al., 2008) that was used as a standard. The asterisk indicates the putative degradation product found for the mutated α3 protein.

We studied the influence of the R1061Q^m^ mutation on the secretion of the single N5 domain and full-length collagen VI and on assembly of large collagen VI tetramers to explore potential mechanisms by which the R1061Q^m^ mutation causes UCMD. Secretion of the single wild-type and mutated N5 domain was tested by recombinant expression in 293 Epstein-Barr nuclear antigen (293EBNA) cells (Figure 6A). The wild-type and the mutated N5 domain were detected in the culture medium and cell extracts in comparable amounts, indicating that the surface exposed missense mutation does not lead to retention and accumulation of the single VWA domain in the secretory pathway.

To determine the influence of the R1061Q^m^ mutation on secretion and assembly of full-length collagen VI, the mutation was introduced into the murine full-length collagen VI α3 chain (N9-C5 region). The wild-type and the mutated constructs were used for transfection of SaOS-2 cells, which produce collagen VI α1 and α2, but not α3, mRNAs (Lamandé et al., 1998). The presence of collagen VI monomers and tetramers in the culture medium and cell lysates of transfected cells was analyzed by electrophoresis in agarose/polyacrylamide composite gels after treatment with 2M urea without reducing agents (Figure 6B). Culture medium of cells with the wild-type and the mutated construct was harvested at different time points. Decreased amounts of mutated collagen VI were detected in the cell culture medium by immunoblot, possibly indicating a slower rate of secretion. However, the formation of tetramers was not perturbed by expression of the mutated α3 chain. At any given time, collagen VI tetramers could be detected for both the wild-type and the mutated protein. Interestingly, in both cell extract and culture medium of cells transfected with the mutated construct, an additional band appeared below the monomers. This probably reflects degradation of the mutated collagen VI protein with time. It remains unclear if this degradation contributes to the drastic phenotype that can be observed in UCMD patients. This disease is often associated with a complete loss of collagen VI protein in the extracellular matrix (Baker et al., 2005). However, the missense mutation may also influence microfibril formation by perturbation of intermolecular interactions or by destabilizing assembled collagen VI proteins. The fact, that it makes a difference if the mutated domain is expressed alone or in context of the full-length collagen VI α3 chain indeed makes it likely that inter- and/or intramolecular interactions of the domain are affected.

### Disease-Associated Mutations of Collagen VI α3 VWA Domains

**Figure 7:**
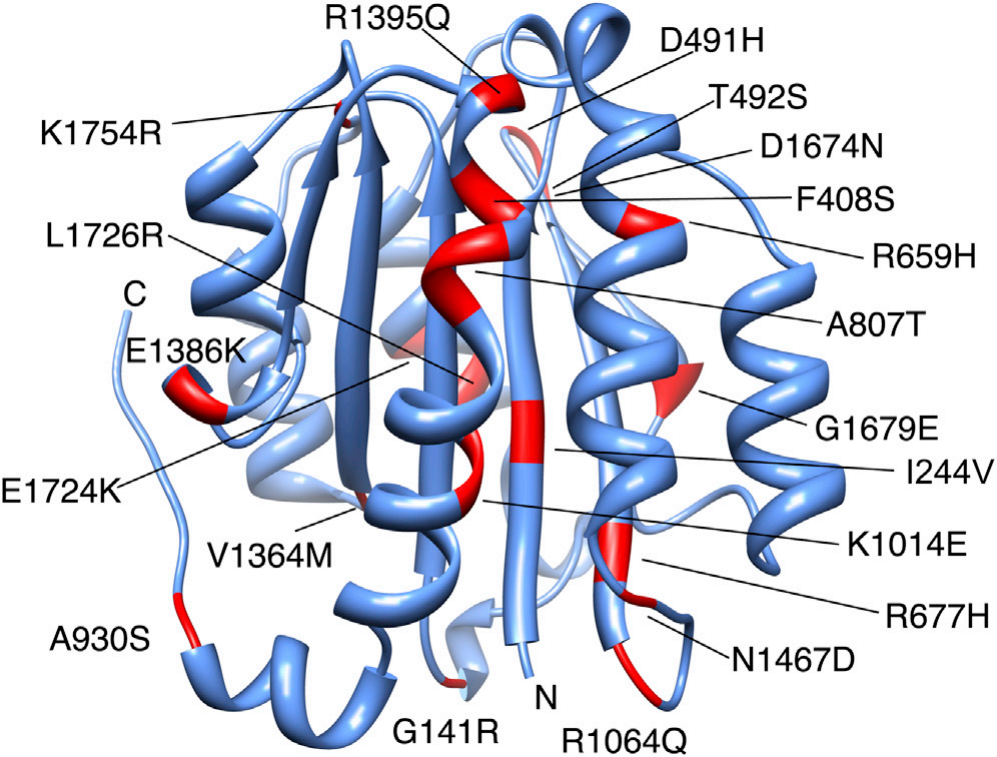
Disease-Causing Mutations Are Mapped onto the Collagen VI α3N5 Domain Structure The murine collagen VI α3N5 structure is shown as a blue ribbon. The locations of mutations from the Leiden database (LOVD; http://www.dmd.nl) as listed in Table 2 are shown in red and labeled according to their sequence position in the full-length human collagen VI α3 protein (ADL14511.1).

The structure of the collagen VI α3N5 domain was used to assess the potential impact of disease-associated mutations (Lampe et al., 2005) (LOVD; http://www.dmd.nl). The point mutations were mapped onto the mouse collagen VI α3N5 domain structure (Figure 7). The mutations are spread throughout the domain, including surface exposed loops, secondary structure elements, as well as a series of positions that are part of the protein core. Homology models of the VWA domains affected by mutations were analyzed for the potential impact of the mutation. Most of the mutated side chains are surface exposed (Table 2), suggesting that the disease-causing effect is related to a perturbation of sites for interactions with other proteins. The solution structure of the N9-N1 construct of the collagen VI α3 chain suggests a well-ordered extended conformation with an overall C shape (Beecher et al., 2011). This implies the presence of a significant number of domain-domain interfaces that could be perturbed by surface mutations. In addition, the supramolecular assembly of the collagen VI tetramer junction (Baldock et al., 2003) suggests further VWA domain interactions between the N-terminal and C-terminal VWA arrays. Furthermore in native collagen VI microfibrils, the domains are associated with other proteins such as decorin, biglycan, and matrilins (Wiberg et al., 2003).

**Table 2:** Disease-Associated Mutations of Human Collagen VI α3 VWA Domains

Domain	Mutation	Location on Structure	Disease	Reference
N10	G141R	surface exposed	unspecified myopathy	LOVD
N9	I244V	buried	unspecified myopathy	LOVD
N8	D491H	surface exposed	unspecified myopathy	LOVD
	T492S	surface exposed	unspecified myopathy	LOVD
	A596S	partially buried	unspecified myopathy	LOVD
N7	R659H	partially buried	unspecified myopathy	LOVD
	R677H	partially buried	BM	Lampe et al., 2005
	A807T	surface exposed	unspecified myopathy	LOVD
	A930S	surface exposed	unspecified myopathy	LOVD
N6	K1014E	surface exposed	BM	Lampe et al., 2005
N5	R1064Q	surface exposed	UCMD	Lampe et al., 2005
N4	E1386K	surface exposed	BM	Lampe et al., 2005
	R1395Q	surface exposed	UCMD	Lampe et al., 2005
	V1364M	buried	unspecified myopathy	LOVD
N3	N1467D	surface exposed	BM	Lampe et al., 2005
N2	D1674N	surface exposed	UCMD	Lampe et al., 2005
	G1679E	buried	BM	Lampe et al., 2005
	E1724K	surface exposed	unspecified myopathy	LOVD
	L1726R	buried	BM	Baker et al., 2007
	K1754R	surface exposed	unspecified myopathy	LOVD

Four mutations affect residues that are buried in the core of their respective domain (Figure 7 and Table 2). Only G1679E and L1726R on the human N2 domain lead to significant perturbations in the packing of the protein core, suggesting a destabilization of the protein fold. Interestingly, in earlier studies, these two mutations were studied using patient samples. The dominant BM-causing G1679E mutation located in the N2 domain of the collagen VI α3 chain segregated to all affected family members in a large pedigree. In cell cultures, the ratios of the α3 to α1 plus α2 chains synthesized by control and patient fibroblasts were similar (Pan et al., 1998). However, in a study where the isolated N2 domain was recombinantly expressed in 293EBNA cells, the secretion of the mutated N2 domain was severely reduced compared to that of the wild-type domain, indicating that the protein does not fold properly and is degraded in the ER (Sasaki et al., 2000). Indeed, our improved model of the N2 domain confirmed that the position of the affected amino acid residue is located in the β2 strand. The exact mechanism remains unclear, but the G1679E mutation may cause disease simply by decreasing the amount of properly folded α3 chains available for collagen VI assembly. It is not clear whether an α3 chain carrying the G1679E mutation would fold at all, as radioimmunoassays showed a 20%–30% loss of α3 chain in patient fibroblast medium and cell lysates (Sasaki et al., 2000). In contrast, the dominant L1726R mutation that was found in a BM patient without affected relatives had no detectable effect on either intracellular or extracellular assembly, and normal amounts of collagen VI were deposited in the extracellular matrix (Baker et al., 2007). In the absence of biochemical characterization of the patient extracellular matrix and because of the highly polymorphic nature of collagen VI genes, it is difficult to establish the pathogenicity of single mutations (Lampe and Bushby, 2005).

The structures reported here show a C-terminal extension creating the first VWA domain with N and C termini at opposing sides of the protein rather than the consensus arrangement for VWA domains that places the termini in close proximity on the same face of the domain. Analysis of a point mutation that is associated with UCMD shows that the structure of the isolated domain that has the mutation is essentially the same as the wild-type, but subtle differences in cellular processing suggest that the mutation affects inter- and/or intramolecular interactions in collagen VI processing or assembly. The implications of these findings are important for the understanding of the overall architecture of VWA domain containing proteins and the genotype phenotype relation in collagen VI associated muscular dystrophies and more generally demonstrates the versatility of domain structures.

## Experimental Procedures

### Protein Expression and Purification

The borders of the collagen VI α3N5 domain were chosen according to the exon sequence. The identity between the murine and the human sequence is 85%. The murine sequence was chosen to be able to take advantage of the mouse as a model organism in future experiments. A DNA fragment encoding the murine collagen VI α3N5 domain with 5′-terminal NheI and 3′-terminal BamHI restriction sites was amplified by PCR using the primers 5′-CTAGCTAGCTCAGGTGAAAAGGATGTGGTG-3′ (forward) and 5′-CGCGGATCCTTAACCTGCACCTGTTGAGGGC-3′ (reverse), respectively. After digestion with NheI and BamHI, the PCR fragment was cloned into the pBluescript II KS (+) vector (Stratgene). This plasmid served as a template for a mutagenesis PCR to insert the R1061Q^m^ mutation using the QuikChange Site-Directed Mutagenesis Kit (Stratagene) according to the manufacturer’s instructions (forward: 5′-GATGTGGGTCCCGACCAGGTGCGTGTGGCA-3′, reverse: 5′-TGCCACACGCACCTGGTCGGGACCCACATC-3′). Wild-type and mutated DNA were digested with NheI and BamHI and cloned into a modified pET 15b expression vector (Novagen) containing an N-terminal One-STrEP-tag followed by a thrombin cleavage site. Both constructs were sequenced and used for transformation of *E. coli* BL21 Rosetta cells (Novagen). Single colonies were cultured overnight at 37°C in 10 ml LB-medium and 50 μg/ml ampicillin for selection. The overnight cultures were used to inoculate 8 l LB-medium containing 50 μg/ml ampicillin and the cultures were grown to A_600_ = 0.4–0.6 at 37°C. Subsequently, protein expression was induced by adding isopropyl-β-D-1-thiogalactopyranoside (IPTG) to a final concentration of 1 mM and the cultures were incubated overnight at 28°C. After harvesting, the cells were resuspended in 50 ml lysis buffer (20 mM Tris [pH 7.4], 150 mM NaCl) and lysed by sonication. Insoluble material was removed by centrifugation at 15,000 rpm and 4°C and the supernatant applied to a Streptactin column (IBA). After washing with lysis buffer, the recombinant protein was eluted with the same buffer containing 2.5 mM desthiobiotin. The One-STrEP-tag was removed by thrombin digestion using a kit (Novagen). Cleaved protein was separated from thrombin and remaining uncleaved material by size exclusion chromatography on a Superdex 75 16/60 column. Peak fractions were pooled, analyzed by SDS-PAGE, and concentrated by ultrafiltration to 14 mg/ml (wild-type) and 15.3 mg/ml (R1061Q^m^), respectively.

### Cell Culture and Transfection

The collagen VI α3N5 domain constructs (wild-type and with R1064Q^m^-mutation [see above]) were cut with NheI and BamHI and inserted into a modified pCEP-Pu vector containing an N-terminal BM-40 signal peptide followed by a One-STrEP-tag (IBA). The murine collagen VI α3 N9-C5 region was generated by PCR and cloned with 5′-terminal SpeI and 3′-terminal NotI restriction sites using the primers 5′-GGACTAGTCTCAGCTGACATTATTTTCCTTATT G-3′ (forward) and 5′-GAATGCGGCCGCTTAAACTGTTAACTCAGGACTACACATC-3′ (reverse). The amplified PCR fragment was ligated into a modified pcDNA6 vector (Invitrogen) containing an N-terminal BM40 signal sequence. To insert the R1061Q^m^ mutation, a cDNA fragment encoding the region between the two internal restriction sites NdeI and SbfI was amplified by PCR using the primers 5′-CCCTAACCACATATGTTAGTGGAG-3′ (forward) and 5′-CCTTAGCTCCTGCAGGGCCCCTTC-3′ (reverse), respectively. This fragment was used as a template for a mutagenesis PCR (see above). The mutated PCR fragment was digested with NdeI and SbfI and ligated into the collagen VI α3 N9-C5 region and subsequently cloned into the modified pcDNA6 vector (Invitrogen). The constructs of the wild-type and mutated N5 domain were used for transfection of 293EBNA cells and the wild-type and mutated α3 constructs were used for transfection of the SaOS-2 human osteosarcoma cell line using FuGENE HD transfection reagent (Promega). Cells were cultured in Dulbecco’s modified Eagle’s medium containing 10% (v/v) fetal calf serum and 0.5 μg/ml puromycin (293EBNA) or 5 μg/ml blasticidin S hydrochloride (SaOS-2) for selection. L-ascorbic acid and L-ascorbic acid 2-phosphate sesquimagnesium salt hydrate were added to SaOS-2 cells every 1–2 days to a final concentration of 0.25 mM and 0.45 mM, respectively.

### SDS-PAGE, Agarose/Polyacrylamide Composite Gel Electrophoresis, and Immunoblotting

Cell extracts and culture medium of transfected and nontransfected 293EBNA cells were reduced with 5% β-mercaptoethanol and subjected to SDS-PAGE on 12% (w/v) SDS-polyacrylamide gels. Cell extracts and culture medium of transfected SaOS-2 cells were supplemented with SDS-containing loading buffer and urea to a final concentration of 2M and subjected to electrophoresis on 0.5% (w/v) agarose/2.4% (w/v) polyacrylamide composite gels (Peacock and Dingman, 1968) under nonreducing conditions to analyze collagen VI assembly. Purified full-length collagen VI protein (a gift from Paolo Bonaldo, Padova) was used as a size standard on composite gels.

For immunoblots, the proteins were transferred to nitrocellulose. After blocking with Tris-buffered saline containing 5% milk powder (single N5 domain) or 1% BSA and 5% milk powder (α3 N9-C5), the nitrocellulose membrane was incubated with an antibody against the strep-tag (gift from Birgit Kobbe) to detect the single N5 domain or an affinity-purified antibody against the murine collagen VI α3 C terminus to detect the α3 N9-C5 region. Bound antibodies were detected by enhanced chemoluminescence using peroxidase-conjugated swine anti-rabbit immunoglobulin G (Dako), 3-aminophthalhydrazide (1.25 mM), p-coumaric acid (225 mM), and 0.01% H_2_O_2_.

### Crystallization and Data Collection

Crystallization experiments were performed at room temperature using the sitting-drop vapor diffusion method. A crystal of collagen VI α3N5 was observed in 0.1 M sodium cacodylate (pH 6.5), 1 M tri-sodium citrate using the 0.2:0.1 ratio, whereas a crystal of the R1061Q^m^ mutant was observed in 0.2 M sodium chloride, 0.1 M phosphate/citrate (pH 4.2), 20% w/v PEG 8000 in the 0.1:0.1 ratio. The crystal of collagen VI α3N5 grew to the dimensions of 170 μm × 100 μm × 50 μm, whereas crystals of R1061Q^m^ grew to the dimensions of 100 μm × 90 μm × 40 μm in 1 week. All crystals were cryoprotected with 30% (v/v) glycerol and flash-frozen in liquid nitrogen for storage. X-ray diffraction data were collected at 100 K using the beamline ID24 at Diamond (Oxford, UK) at a wavelength of λ = 0.97780 with a Pilates 6M detector.

A complete data set of collagen VI α3N5 was collected to 1.2 Å resolution (0.15° oscillation with 0.2 s exposure time). The data were integrated and processed with iMOSFLM (Battye et al., 2011) from the CCP4 (CCP4, 1994) suite. The crystal belongs to the space group P2_1_ and had unit cell dimensions of a = 37.7, b = 58.6, c = 39.3, alpha = 90°, beta = 113°, gamma = 90°. The Mathews coefficient is 1.74 and the solvent content is 29.4% with one molecule in the asymmetric unit.

A complete data set of R1061Q^m^ was collected to 1.2 Å resolution (0.15° oscillation with 0.1 s exposure time). The data were integrated and processed with XIA (Winter, 2010). The crystal belongs to the space group P4_3_2_1_2 and had unit cell dimensions a = 55.1, b = 55.1, c = 106.9, alpha = 90°, beta = 90°, gamma = 90°. The Mathews coefficient is 1.77 and the solvent content is 30.7% with one molecule in the asymmetric unit.

### Structure Determination and Refinement

The structure of VWF A2 (Protein Data Bank code 3GXB) was modified and all side chain residues pruned to Cβ atoms using the program CHAINSAW in CCP4. The output was used as a search model in PHASER (McCoy et al., 2007) for the collagen VI α3N5 data set. The structure was built in parts with ARP/wARP (Langer et al., 2008) and completed with COOT (Emsley and Cowtan, 2004). The initial refinements were carried out using data to 1.5 Å in PHENIX (Adams et al., 2011). Further refinement rounds were performed using diffraction data to 1.2 Å and manual rebuilding. Solvent molecules and alternative conformations were gradually added. A final round of refinement included anisotropic data.

The mutant structure R1061Q^m^ mutant was solved using PHASER with the fully refined native structure of collagen VI α3N5. The structure was refined with PHENIX and model built with COOT (Emsley and Cowtan, 2004). Table 1 summarizes data collection and refinement statistics. Both crystal structures were validated with Molprobity (Chen et al., 2010). The Ramachandran plot for the collagen VI α3N5 structure (R1061Q^m^) shows that 92.1 (93.2) % of non-Gly and non-Pro residues are in the most favored regions and 6.7 (5.6) % are in the additionally allowed regions. The remaining 1.2% is due to two residues, R1061 and S1070, which are in the generously allowed regions.

### Homology Modeling

Homology modeling of the human collagen VI VWA domains was carried out using Modeler 4.12 (Sali and Blundell, 1993) in UCSF Chimera 1.6.1 (Pettersen et al., 2004) using the mouse collagen VI α3N5 as the template structure. The modeling was restricted to the N-terminal domains, N10 to N2, from collagen VI α3, because the sequence identity of the template sequence was sufficiently high (>29%). Somewhat lower sequence identities were observed for the C-terminal domains and VWA domains from other collagen VI chains. For each domain, five models were generated and the one with the lowest normalized discrete optimized protein energy (zDOPE) score was retained for energy minimization. zDOPE scores were less than −1.0 and a small number of clashes suggested reasonable protein structures. The rmsds with the target structure were less than 0.6 Å, indicating a close resemblance of modeled VWA domains with the collagen VI α3N5 domain. The point mutations listed in Table 2 were introduced in the respective modeled domains and their surface exposure and packing inspected. The mutations were classified as either surface exposed, partially buried, or buried depending on whether the side chains of the mutant residue were fully exposed to the solvent or not. In all cases, the mutations of buried residues introduced significant packing perturbations, suggesting that they would perturb the fold of the respective domain.

### Sequence Alignment

Sequences were downloaded from the ENSEMBL mouse genome browser (GRCm38). For the alignments, the complete preceding VWA domain, the linker region, and the beginning of the next VWA domain were used. Multiple sequence alignments were performed using the PILEUP program of the WISCONSIN PACKAGE version 10.3 (Accelrys), using the parameters GapWeight: 6 and GapLengthWeight: 1 for Figure 5A and GapWeight: 2 and GapLengthWeight: 1 for Figure 5B. Figures were prepared with the BOXSHADE v3.21 program. In the figure, only the linker region and adjacent sequences are shown.

### Graphical Representation of Structures

Figures 2, 3, 4, and 7 were generated using UCSF Chimera 1.6.1 (Pettersen et al., 2004).

## Accession Numbers

The coordinates and the structure factors of murine collagen VI α3N5 and R1061Q have been deposited in the RCSB Protein Data Bank with accession numbers 4IGI and 4IHK, respectively.
